# ETV6-RUNX1 and RUNX1 directly regulate RAG1 expression: one more step in the understanding of childhood B-cell acute lymphoblastic leukemia leukemogenesis

**DOI:** 10.1038/s41375-021-01409-9

**Published:** 2021-09-17

**Authors:** Hélène Jakobczyk, Yan Jiang, Lydie Debaize, Benoit Soubise, Stéphane Avner, Aurélien A. Sérandour, Jérémie Rouger-Gaudichon, Anne-Gaëlle Rio, Jason S. Carroll, Hana Raslova, David Gilot, Ziling Liu, Jocelyne Demengeot, Gilles Salbert, Nathalie Douet-Guilbert, Laurent Corcos, Marie-Dominique Galibert, Virginie Gandemer, Marie-Bérengère Troadec

**Affiliations:** 1 Univ Rennes 1, CNRS, IGDR (Institut de génétique et développement de Rennes) - UMR 6290, Rennes, France; 2 Univ Brest, Inserm, EFS, UMR 1078, GGB, Brest, France; 3 Department of Hematology, The First Hospital of Jilin University, Changchun, China; 4 Université de Nantes, Ecole Centrale de Nantes, Inserm, CRCINA, Nantes, France; 5 Department of Pediatric Oncology and Hematology, University Hospital, Caen, France; 6 Cancer Research UK Cambridge Institute, University of Cambridge, Cambridge, UK; 7 INSERM, UMR 1287, Gustave Roussy, Université Paris Saclay, Villejuif, France; 8 Equipe labellisée Ligue Nationale contre le Cancer, Villejuif, France; 9 INSERM, Université Rennes, CLCC Eugène Marquis, UMR_S 1242, Rennes, France; 10 Cancer Center, The First Hospital of Jilin University, Changchun, China; 11 Instituto Gulbenkian de Ciência, Rua da Quinta Grande, 6, Oeiras, Portugal; 12 CHRU Brest, Service de génétique, laboratoire de génétique chromosomique, Brest, France; 13 Centre Hospitalier Universitaire de Rennes (CHU-Rennes), Service de Génétique et Génomique Moléculaire, Rennes, France; 14 Centre Hospitalier Universitaire de Rennes (CHU-Rennes), Department of pediatric hemato-oncology, Rennes, France

**Keywords:** Leukaemia, Haematological diseases

## Abstract

- ETV6-RUNX1 and RUNX1 directly promote *RAG1* expression.
- ETV6-RUNX1 and RUNX1 preferentially bind to the −1200 bp enhancer of *RAG1* and the −80 bp promoter of *RAG1* gene respectively, and compete for these bindings.
- ETV6-RUNX1 and RUNX1 induce an excessive RAG recombinase activity.
- ETV6-RUNX1 participates directly in two events of the multi-hit ALL leukemogenesis: as an initiating event and as an activator of *RAG1* expression.

**To the editor**

Leukemia usually requires multiple genetic events, as exemplified in *ETV6-RUNX1* B-cell precursor acute lymphoblastic leukemia (BCP-ALL), one of the most frequent pediatric BCP-ALL [1]. The translocation t(12;21)(p13;q22) resulting in *ETV6-RUNX1* fusion gene arises predominantly *in utero* and produces a preleukemic clone. Additional mutations occur years after the translocation t(12;21) and give rise ultimately to leukemia [1]. Those additional genetic alterations observed in *ETV6-RUNX1* BCP-ALL are predominantly caused by illegitimate genomic rearrangements mediated by aberrant RAG recombinase activity [2].

The RAG recombinase consists of two subunits, RAG1 and RAG2. It recognizes and cleaves DNA at recombination signal sequence (RSS), and is responsible for the V(D)J rearrangement of immunoglobulin genes during differentiation of B and T lymphoid lineages. Illegitimate off-target RAG cleavages can be pathological. High incidence of recombination events, RAG recombinase aberrant activity and high *RAG1* gene expression have been repeatedly reported in *ETV6-RUNX1* leukemia or equivalent mouse models [3–8]. Consistent with epidemiological findings on childhood BCP-ALL etiology [1], this aberrant RAG recombinase activity can be explained by an excessive immune response or repeated exposure to inflammatory stimuli (chronic infection) [7, 1]. However, a genetic cause of RAG increased activity related to the presence of the fusion gene *ETV6-RUNX1* can also be proposed [2, 3, 7].

A proper regulation of *RAG1* and *RAG2* gene expression is crucial for the integrity of lymphocyte development. This regulation is complex and tightly controlled by promoters, and proximal and distal *cis-*regulatory elements. In B-cells, *Rag1* and *Rag2* genes are controlled by the strong −22kb *Erag* enhancer, the *Irag2* enhancer, *Rag2* distal and proximal enhancers (Ed and Ep), *Rag1* and *Rag2* promoters and *Irag1* located about 15 kb upstream of the *Rag1* promoter. Runx1 is described to be an essential regulator of the *Rag1* promoter and *Rag1-Rag2* silencer and antisilencer in mouse T-cells.

We aimed to delineate the causative link between the presence of RUNX1, the ETV6-RUNX1 fusion protein and *RAG1* upregulation in *ETV6-RUNX1* BCP-ALL. Our findings fulfill a missing step in the multi-hit model of ETV6-RUNX1-related leukemogenesis between the *ETV6-RUNX1* fusion gene and RAG1 aberrant activity.

*RAG1* and *RUNX1* transcripts levels are positively and significantly correlated exclusively in *ETV6-RUNX1* BCP-ALL compared to other childhood BCP-ALL (Fig. 1A, Supplementary Fig. S1A), suggesting either a common regulator for *RAG1* and *RUNX1 (or ETV6-RUNX1)* expression, or a dependency between them.

**Fig. 1:**
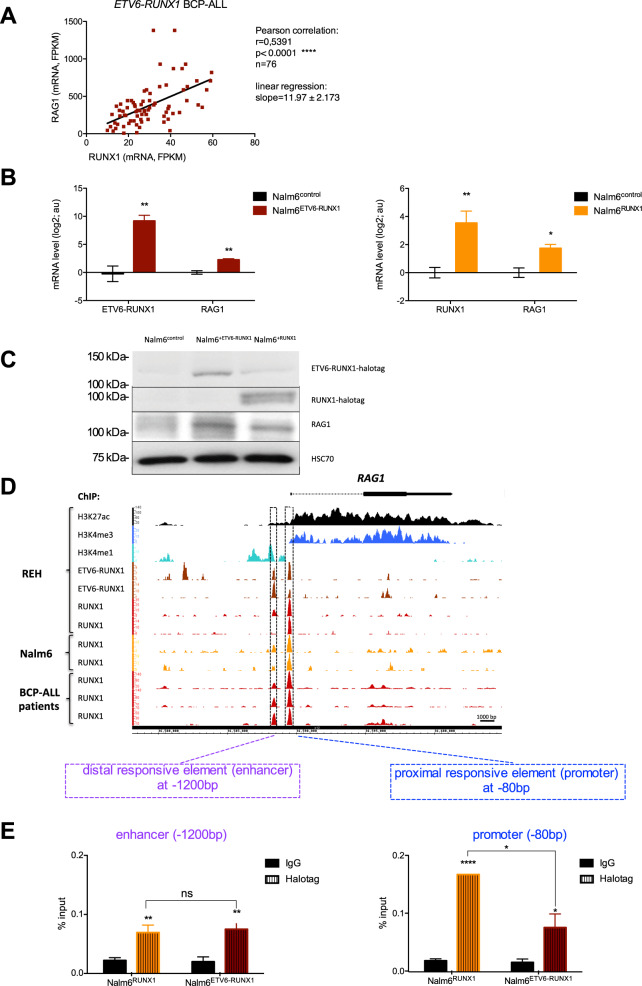
ETV6-RUNX1 and RUNX1 upregulate the expression of *RAG1* mRNA and protein and bind promoter and enhancer of *RAG1* gene. **A** Statistical analysis of the expression between *RUNX1* and *RAG1* mRNA originating from *ETV6-RUNX1* BCP-ALL cells using Pearson correlation. Data of mRNA levels (expressed in Fragments Per Kilobase Million – FPKM) have been extracted from the St. Jude Children’s Research Hospital RNA-Seq Pediatric Cancer Data Portal. **B** Relative mRNA expression of *ETV6-RUNX1*, *RAG1* and *RUNX1* measured by RT-qPCR in Nalm6^control^, Nalm6^ETV6-RUNX1^ and Nalm6^RUNX1^ cells. Results are presented in-terms of a fold change in log2 scale after normalizing with *ABL* mRNA. Each value represents the mean ± S.D. of four independent experiments (i.e. independent stable cell lines). **C** Representative images of western blot showing endogenous RAG1 protein and HSC70 protein (for normalization) in Nalm6^control^, Nalm6^ETV6-RUNX1^ and Nalm6^RUNX1^ cells. The western blot also shows the presence of ETV6-RUNX1 and RUNX1 revealed with Halotag antibody. **D** ChIP-Seq profiles across the human *RAG1* gene. Genomic tracks display ChIP-Seq profiles for RUNX1, ETV6-RUNX1 and the histones H3K4me3, H3K27ac and H3K4me1 from REH cells (2 replicates for RUNX1 and ETV6-RUNX1). RUNX1 ChIP-Seq (2 replicates) for Nalm6 cells and bone marrow mononuclear cells isolated from three pre-B acute lymphoblastic leukemia patients (BCP-ALL) are also displayed. ChIP-Seq data were acquired by Illumina sequencing and visualized with Integrated Genome Browser 9.0.0. ChIP-Seq reads were aligned to the reference human genome version GRCh37 (hg19). Both genomic regions of *RAG1* gene that were associated with an overlap of RUNX1 and ETV6-RUNX1 peaks are indicated by boxes: one region is an enhancer at −1200 bp from TSS, and the other is a promoter at −80 bp from TSS. **E** ChIP-qPCR on the −1200 bp enhancer and the −80 bp promoter with IgG and Halotag antibodies in Nalm6^RUNX1^ and Nalm6^ETV6-RUNX1^ cells. Results are expressed as percentage of input (*n* = 3). TSS: transcription starting site.

To investigate the effect of RUNX1 and ETV6-RUNX1 fusion protein on *RAG1* expression, we used human B-cell precursor Nalm6 cells (see supplemental data for detailed materials and methods, Supplementary Tables S1–S2), which do not normally express the *ETV6-RUNX1* fusion gene. Enforced expression of ETV6-RUNX1 or RUNX1 in Nalm6 cells (named Nalm6^ETV6-RUNX1^ and Nalm6^RUNX1^) induced high levels of endogenous *RAG1* transcript and protein (Fig. 1B–C, Supplementary Fig. S1B). Expression of an inactive RUNX1 (due to a truncation in the RUNT DNA-binding domain) decreases the expression of *RAG1* transcript and protein compared to wild-type RUNX1 (Supplementary Fig. S1C–D). Those results demonstrated that both ETV6-RUNX1 and RUNX1 upregulate, directly or indirectly, the expression of *RAG1* and, additionally, that the DNA-binding domain of RUNX1 is involved in this regulation.

To investigate whether ETV6-RUNX1 and RUNX1 could be recruited to the *RAG* locus control region in human pre-B lymphocytes, we performed chromatin immunoprecipitation followed by sequencing (ChIP-Seq) with RUNX1 and ETV6 antibodies in different cells: bone marrow mononuclear cells from 3 childhood BCP-ALL patients negative for ETV6-RUNX1, Nalm6 cells, and REH cells that express ETV6-RUNX1 fusion protein (Supplementary Fig. S2). Of note, REH cells are deleted for the normal *ETV6* allele; ChIP-Seq with ETV6 antibody in REH cells is specific for ETV6-RUNX1. We have already described the genomic occupancy of RUNX1 in BCP-ALL patients, Nalm6 and REH cells [9, 10]. Additionally, RUNX1 and ETV6-RUNX1 share 5,377 peaks in REH cells, and about 2000 peaks are uniquely identified for ETV6-RUNX1 (Supplementary Fig. S2A). About 25% of the regions occupied by RUNX1 or ETV6-RUNX1 are transcriptionally active (H3K4me1, markers of active enhancers; H3K4me3, active promoters; H3K27ac, transcriptionally active chromatin) (Supplementary Fig. S2B). Several peaks for RUNX1 and ETV6-RUNX1 are observed within the + /−100kb region overlapping the *RAG* locus control region (Supplementary Fig. S2C). Two peaks were clearly identified at −1200 bp (referred to enhancer because of its negativity for H3K4me3 and positivity for H3K4me1) and −80 bp (promoter, H3K4me3 positive, H3K4me1 negative) from *RAG1* transcription starting site (TSS), in all the samples (BCP-ALL patients, Nalm6 and REH cells) and shared for RUNX1 and ETV6-RUNX1 (Fig. 1D, Supplementary Fig. S2C).

In RUNX1 ChIP-seq profiles from Nalm6 and BCP-ALL patients’ cells, RUNX1 seems to preferentially bind the −80 bp proximal region compared to the −1200 bp. We confirmed this preferential binding of RUNX1 on the −80 bp proximal region compared to the −1200 bp enhancer by ChIP-qPCR (Supplementary Fig. S3A). We also observed that ETV6-RUNX1 binds to the −1200 bp enhancer similarly to RUNX1 (Fig. 1E, left), but less than RUNX1 on the −80 bp promoter (Fig. 1E, right). Competition assays between tagged RUNX1 and ETV6-RUNX1 on each of these two regulatory regions showed that ETV6-RUNX1 is a potent competitor for the binding to −1200 bp *RAG1* enhancer while RUNX1 is the major binding protein for −80 bp *RAG1* promoter (Supplementary Fig. S3B–E).

To ascertain the physiological role of these regions on the regulation of *RAG1* expression, we used a CRISPR-mediated activation system dCas9-VP64, where dCas9 is dead and VP64 induces transcription [11]. In HEK cells, the three gRNAs targeting the −1200 bp enhancer did not significantly affect *RAG1* transcript level, probably due to some limitation of this technique to achieve a long-range action [11, 12] (Supplementary Fig. S4A). On the contrary, a strong activation of *RAG1* mRNA expression is observed with gRNAs targeting the −80 bp *RAG1* promoter in HEK cells and Nalm6 cells, an appropriate model for BCP-lymphoblasts (Fig. S4A, Supplementary Fig. 2A). We also observed a slight increase in RAG1 protein level (Fig. 2B). Altogether, those results demonstrate that the −80 bp *RAG1* promoter is a physiologically active site of transcription in pre-B cells and controls *RAG1* expression.

**Fig. 2:**
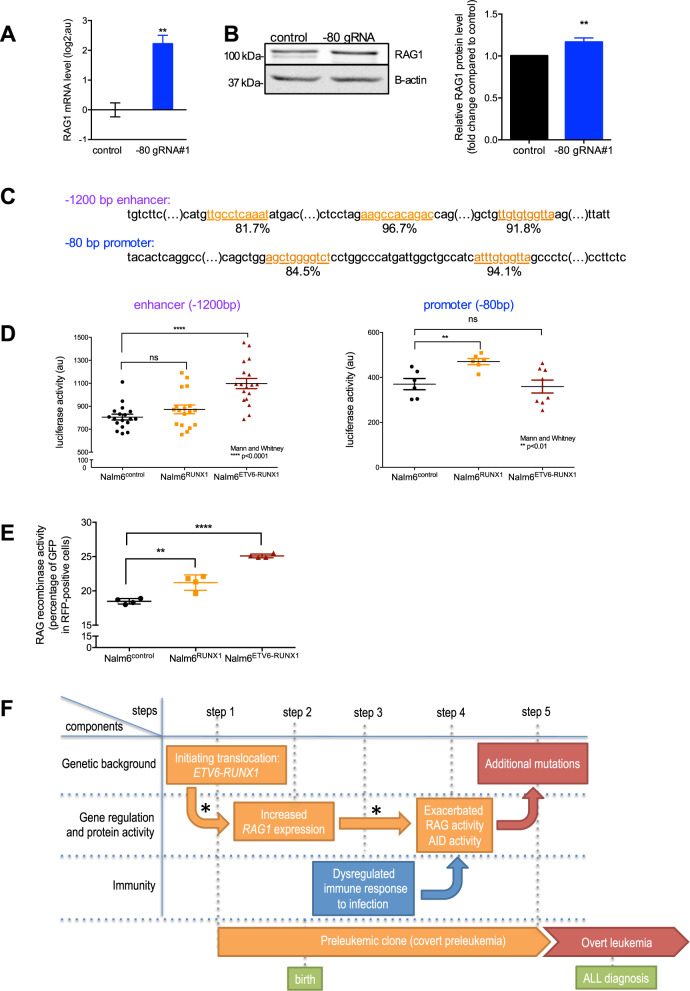
ETV6-RUNX1 and RUNX1 physiologically activate the transcription of *RAG1* and increase RAG-mediated recombination. **A** Relative mRNA expression of *RAG1* measured by RT-qPCR in Nalm6^control^ and Nalm6^−80gRNA^ cells for the CRISPR dCas9-VP64 experiments. The gRNA used correspond to gRNA#1 for the −80 bp promoter. Results are presented in-terms of a fold change in log2 scale after normalizing with *GAPDH* mRNA. Each value represents the mean ± S.D. of three independent experiments (i.e. independent stable cell lines). **B** Representative images of western blot (left panel) and densitometric analysis (right panel) showing the quantitation of endogenous RAG1 protein in Nalm6^control^ and Nalm6^−80gRNA^ cells. Results are presented after normalizing with β-actin protein levels. Each value represents the mean ± S.D. of three independent experiments. **C** Partial sequences of the −1200 bp enhancer and the −80 bp promoter with indication of putative RUNX1 binding sequence (identified *in silico* by JASPAR). The relative score is indicated below each sequence. **D** Luciferase assays with plasmids containing either the −1200 bp enhancer (left panel) or the −80 bp promoter (right panel) of *RAG1* upstream a minimal promoter and a luciferase ORF in Nalm6^control^, Nalm6^RUNX1^ and Nalm6^ETV6-RUNX1^ cells. Luciferase levels are represented using a scatter dot plot indicating the means and S.D. of at least 4 experiments. ns: non-significant. **E** Quantification, by flow cytometry, of RAG-mediated recombination using the reporter assay from [15] in Nalm6^control^, Nalm6^RUNX1^ and Nalm6^ETV6-RUNX1^ cells, 7 days after transduction with GFPi vectors. Absence of RAG activity results in only RFP (red fluorescence protein) production, while active RAGs mediate the inversion of the GFP (green fluorescence protein) gene allowing for GFP and RFP production. Results are expressed as the percentage of GFP-positive cells in RFP-positive cells (*n* = 4). **F** Proposed schematic representation of the ETV6-RUNX1 “multi-hit” leukemogenesis model with a causative transcriptional link between the first and second steps. The * indicates the steps demonstrated in this work. AID: activation-induced cytidine deaminase.

Next, we validated the specific binding sites of RUNX1 predicted *in silico* by JASPAR in HEK cells by deleting those sites in luciferase assays (Fig. 2C, Supplementary Fig. S4B). As expected in non-hematopoietic lineages ETV6-RUNX1 is not active on those luciferase assays [13, 14]. Importantly, we demonstrated the responsiveness of those two regulatory regions on *RAG1* expression in pre-B cells using the corresponding retroviral-luciferase reporter assay in Nalm6 cells (Fig. 2D). Stable expression of ETV6-RUNX1 in Nalm6 cells induces luciferase activity with the −1200 bp enhancer, whereas stable overexpression of RUNX1 activates the −80 bp proximal promoter. This result demonstrates that, in a proper B-cell lineage, the −1200 bp enhancer is activated by ETV6-RUNX1, and that RUNX1 is a major activator of the −80 bp promoter.

We next tested the link of causality between the expression of ETV6-RUNX1 or RUNX1 and the increase in RAG recombinase activity, making use of the quantitative GFPi reporter assay [15]. In this assay, the higher the GFP signal, the higher the RAG recombinase activity. When applied to Nalm6^RUNX1^ and Nalm6^ETV6-RUNX1^ cells, the GFPi assay shows value significantly higher than for Nalm6^control^, demonstrating that enforced expression of RUNX1 as well as ETV6-RUNX1 causes an increase in RAG activity in pre-B lymphoblasts (Fig. 2E, Supplementary Fig. S4C).

Taken together, these data contribute to complete the multi-hit model of *ETV6-RUNX1* BCP-ALL leukemogenesis. We propose the following model that involves components from genetics, gene expression and activity, and immunity (Fig. 2F). The first step consists in the t(12;21)(p13;q22) translocation that usually occurs *in utero* and produces the *ETV6-RUNX1* fusion gene. For step 2, the abnormal ETV6-RUNX1 transcription factor and RUNX1 directly induce *RAG1* overexpression by binding mainly to the −1200 bp enhancer and the −80 bp promoter in the *RAG1* locus, respectively. For step 3, a dysregulated immune response occurs during infections [1]. The binding of ETV6-RUNX1 and RUNX1 to the *RAG1* locus (step 2) results in a *RAG* aberrant increased activity (step 4), and participates, together with additional stimuli such as inflammation and abnormal immune response (step 3) in the generation of inappropriate genomic rearrangements (step 5) [2, 7, 1]. Those additional mutations will promote conversion of the ETV6-RUNX1 preleukemic clone into overt leukemia.

The involvement of ETV6-RUNX1 in the upregulation of RAG1 has been previously examined in vitro and in murine preleukemic *ETV6-RUNX1* pro/pre B cells [7, 8]. However, our data, while concordant with those results, go beyond. We demonstrate a direct causal hierarchy between the presence of ETV6-RUNX1 and RUNX1 proteins and RAG1 upregulation. Our findings clearly demonstrate that *RAG1* transcripts are directly upregulated by ETV6-RUNX1 from the –1200 bp *RAG1* enhancer and by RUNX1 from the –80 bp *RAG1* promoter in human pre-B cells. Our findings are complementary to previous studies unraveling the infectious/immune component of *ETV6-RUNX1* BCP-ALL. Those reports demonstrated that the activation-induced cytidine deaminase (AID) and/or RAG recombinase drove leukemia with repeated exposure to inflammatory stimuli (step 3 of our model), paralleling chronic infections in childhood [7, 8]. We demonstrated that ETV6-RUNX1 and RUNX1 directly induce *RAG1* overexpression (step 2) and a direct link between *RAG1* overexpression and *RAG* aberrant increased activity (step 2 and step 4).

We propose a convincing model directly linking the leukemia-initiating event (i.e., the t(12;21) *ETV6-RUNX1* translocation) with upregulation of *RAG1* as well as with a stronger activity of RAG recombinase as observed in *ETV6-RUNX1* BCP-ALL leukemogenesis.

## Supplementary information

Figure S1

Figure S2

Figure S3

Figure S4

supplemental information

## References

[1] Greaves M. A causal mechanism for childhood acute lymphoblastic leukaemia. Nat Rev Cancer. 2018;18:471. 10.1038/s41568-018-0015-6 PMC6986894 29784935

[2] Papaemmanuil E, Rapado I, Li Y, Potter NE, Wedge DC, Tubio J (2014). RAG-mediated recombination is the predominant driver of oncogenic rearrangement in ETV6-RUNX1 acute lymphoblastic leukemia. Nat Genet.

[3] Hübner S, Cazzaniga G, Flohr T, van der Velden VHJ, Konrad M, Pötschger U (2003). High incidence and unique features of antigen receptor gene rearrangements in TEL–AML1-positive leukemias. Leukemia.

[4] Waanders E, Scheijen B, van der Meer LT, van Reijmersdal SV, van Emst L, Kroeze Y (2012). The origin and nature of tightly clustered BTG1 deletions in precursor B-cell acute lymphoblastic leukemia support a model of multiclonal evolution. PLoS Genet.

[5] Zhang M, Swanson PC (2008). V(D)J Recombinase binding and cleavage of cryptic recombination signal sequences identified from lymphoid malignancies. J Biol Chem.

[6] Ross ME, Zhou X, Song G, Shurtleff SA, Girtman K, Williams WK (2003). Classification of pediatric acute lymphoblastic leukemia by gene expression profiling. Blood.

[7] Swaminathan S, Klemm L, Park E, Papaemmanuil E, Ford A, Kweon S-M (2015). Mechanisms of clonal evolution in childhood acute lymphoblastic leukemia. Nat Immunol.

[8] Rodríguez-Hernández G, Hauer J, Martín-Lorenzo A, Schäfer D, Bartenhagen C, García-Ramírez I (2017). Infection exposure promotes ETV6-RUNX1 precursor B-cell leukemia via impaired H3K4 demethylases. Cancer Res.

[9] Debaize L, Jakobczyk H, Avner S, Gaudichon J, Rio A-G, Sérandour AA (2018). Interplay between transcription regulators RUNX1 and FUBP1 activates an enhancer of the oncogene c-KIT and amplifies cell proliferation. Nucleic Acids Res.

[10] Jakobczyk H, Debaize L, Soubise B, Avner S, Rouger-Gaudichon J, Commet S (2021). Reduction of RUNX1 transcription factor activity by a CBFA2T3-mimicking peptide: application to B cell precursor acute lymphoblastic leukemia. J Hematol OncolJ Hematol Oncol.

[11] Konermann S, Brigham MD, Trevino AE, Joung J, Abudayyeh OO, Barcena C (2015). Genome-scale transcriptional activation by an engineered CRISPR-Cas9 complex. Nature.

[12] Li K, Liu Y, Cao H, Zhang Y, Gu Z, Liu X (2020). Interrogation of enhancer function by enhancer-targeting CRISPR epigenetic editing. Nat Commun.

[13] Hiebert SW, Sun W, Davis JN, Golub T, Shurtleff S, Buijs A (1996). The t(12;21) translocation converts AML-1B from an activator to a repressor of transcription. Mol Cell Biol.

[14] Fears S, Gavin M, Zhang DE, Hetherington C, Ben-David Y, Rowley JD (1997). Functional characterization of ETV6 and ETV6/CBFA2 in the regulation of the MCSFR proximal promoter. Proc Natl Acad Sci USA.

[15] Trancoso I, Bonnet M, Gardner R, Carneiro J, Barreto VM, Demengeot J, et al. A Novel Quantitative Fluorescent Reporter Assay for RAG Targets and RAG Activity. Front Immunol [Internet]. 16 mai 2013 [cité 4 avr 2019];4. Disponible sur: https://www.ncbi.nlm.nih.gov/pmc/articles/PMC3655321/. 10.3389/fimmu.2013.00110 PMC3655321 23720659

