# Supplementary figures and images for: ETV6-RUNX1 and RUNX1 directly regulate RAG1 expression: one more step in the understanding of childhood B-cell acute lymphoblastic leukemia leukemogenesis
Source: Leukemia. 2021 Sep 17;36(2):549–54. doi: 10.1038/s41375-021-01409-9 (PMC8807389; doi:10.1038/s41375-021-01409-9)

# Figure S1

**A**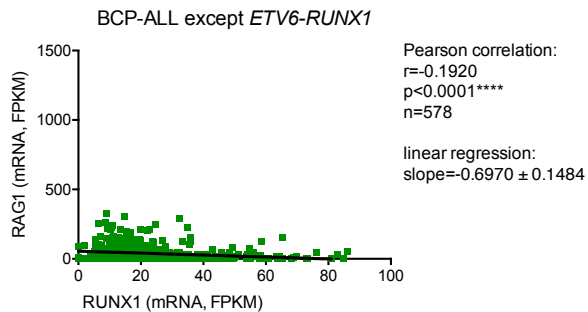**B**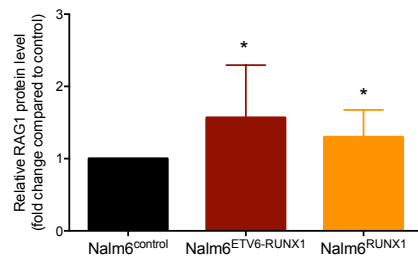**C**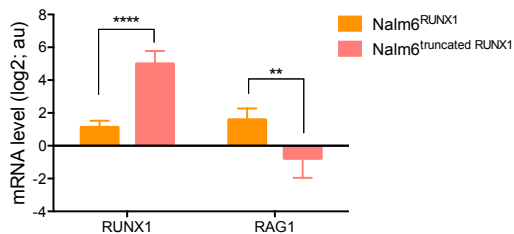**D**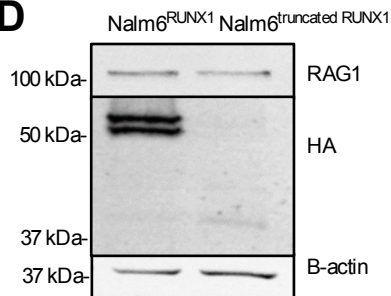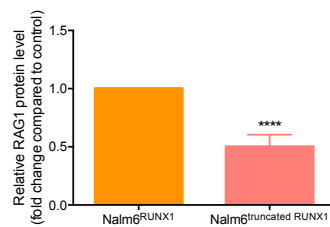

Supplement: Supplementary file 1 — Figure S1 [file 41375_2021_1409_MOESM1_ESM.pdf]

# Figure S2

**A**

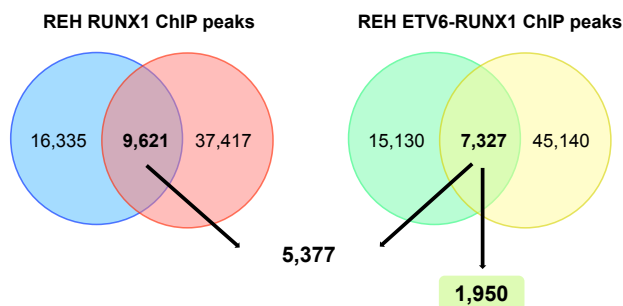

**B**

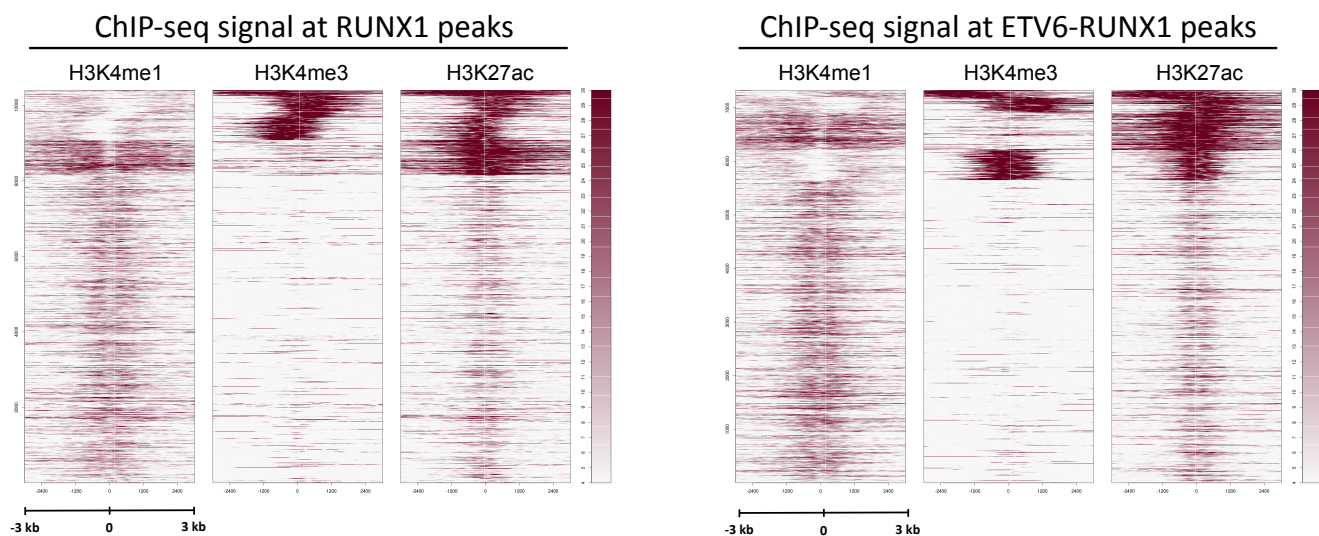

**C**

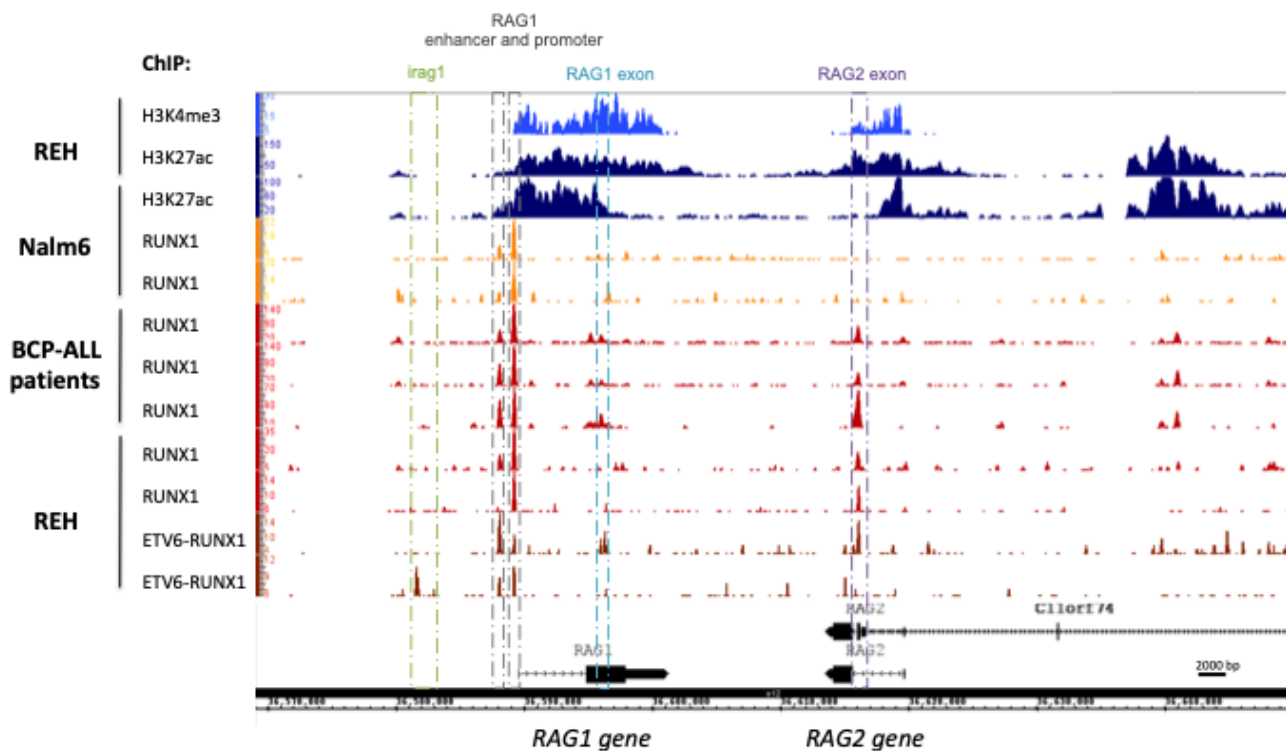

Supplement: Supplementary file 2 — Figure S2 [file 41375_2021_1409_MOESM2_ESM.pdf]

# Figure S3

## A

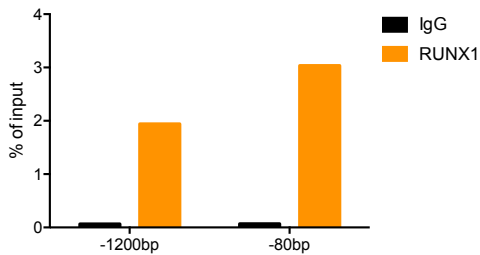

## B

enhancer (-1200bp)

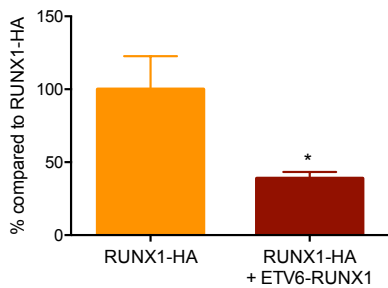

## C

enhancer (-1200bp)

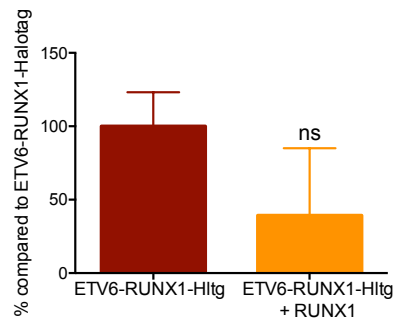

## D

promoter (-80bp)

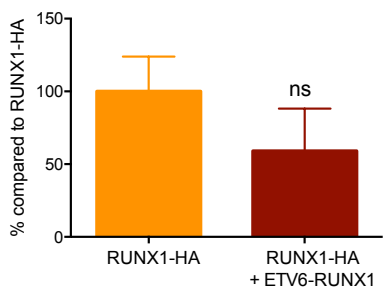

## E

promoter (-80bp)

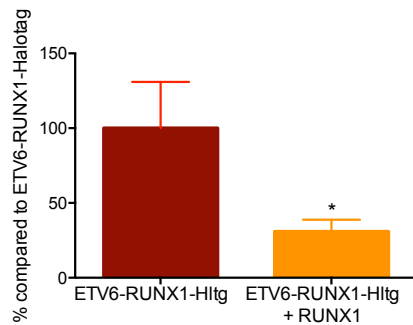

Supplement: Supplementary file 3 — Figure S3 [file 41375_2021_1409_MOESM3_ESM.pdf]

# Figure S4

**A**

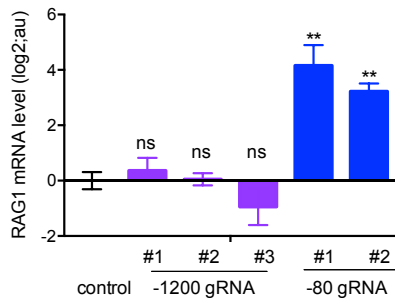

**B**

enhancer (-1200bp)

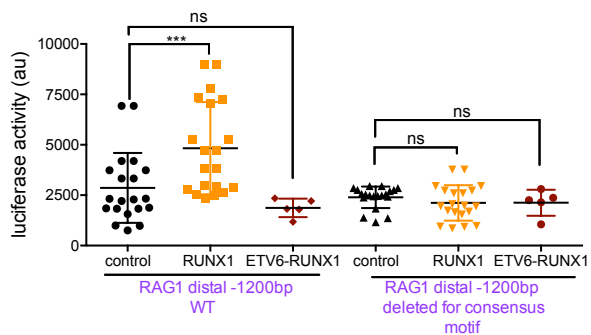

promoter (-80bp)

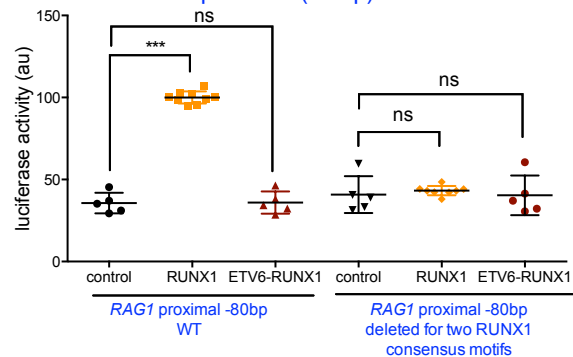

**C**

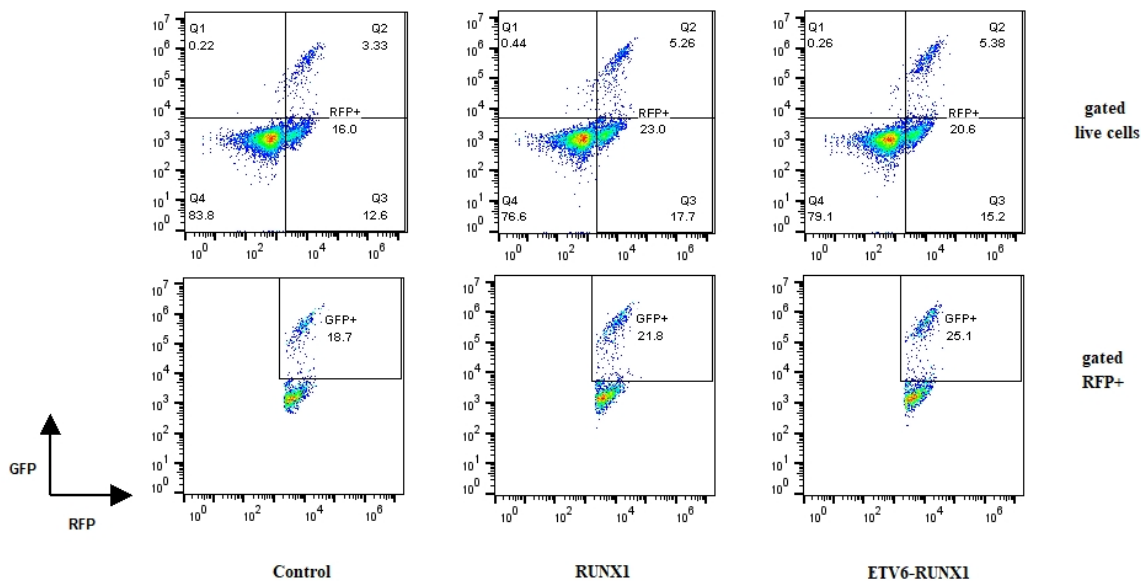

Supplement: Supplementary file 4 — Figure S4 [file 41375_2021_1409_MOESM4_ESM.pdf]
