# Supplementary material for: ETV6-RUNX1 and RUNX1 directly regulate RAG1 expression: one more step in the understanding of childhood B-cell acute lymphoblastic leukemia leukemogenesis
Source: Leukemia. 2021 Sep 17;36(2):549–54. doi: 10.1038/s41375-021-01409-9 (PMC8807389; doi:10.1038/s41375-021-01409-9)
Supplement: Supplementary file 5 — supplemental information [file 41375_2021_1409_MOESM5_ESM.docx]

ETV6-RUNX1 AND RUNX1 DIRECTLY REGULATE RAG1 EXPRESSION : ONE MORE STEP IN THE UNDERSTANDING OF ETV6-RUNX1 CHILDHOOD B-ACUTE LYMPHOBLASTIC LEUKEMIA LEUKEMOGENESIS

By Hélène Jakobczyk^1#^, Yan Jiang^2,3#^, Lydie Debaize^1^, Benoit Soubise^2^, Stéphane Avner^1^, Aurélien A. Sérandour^4^, Jérémie Rouger-Gaudichon^5^, Anne-Gaëlle Rio^1^, Jason S. Carroll^6^, Hana Raslova^7^, David Gilot^8^, Ziling Liu^9^, Jocelyne Demengeot^10^, Gilles Salbert^1^, Nathalie Douet-Guilbert^2,11^, Laurent Corcos^2^, Marie-Dominique Galibert^1,12^, Virginie Gandemer^1,13^, Marie-Bérengère Troadec^1,2,11*^

***SUPPLEMENTAL MATERIAL AND METHODS***

**Cell lines, patients’ cells and plasmids**

Pre-B cell lines, REH, Nalm6^control^, Nalm6^ETV6-RUNX1^ and Nalm6^RUNX1^ stable cell lines were previously described[1]. HA-RUNX1 and truncated HA-RUNX1-R139X (truncation in the RUNT domain) were cloned into the pRRL-EF1α_MCS-PGK-GFP lentiviral vector as previously described [2]. BCP-ALL bone marrow mononuclear cells were collected at diagnosis after informed consent, in accordance with the declaration of Helsinki, and prepared as described[3]. The protocol was approved by the ethics committee of Rennes Hospital (France).

**RNA extraction, cDNA synthesis, RT-qPCR, and Western blot**

RNA was extracted using the NucleoSpin RNA plus (Macherey Nagel). cDNA was synthesized using High-capacity cDNA RT kit (Life Technologies) according to the manufacturer's protocol. Real-time PCR was carried out in sealed 96-well microtiter plates using the SYBR™ Green PCR Master Mix (Applied Biosystems), according to Applied Biosystems gene amplification specifications (40 cycles of 15 sec at 95°C and 1 min at 60°C). The forward (F) and reverse (R) primers were described in **Table S1.** Data analysis was performed using the ΔΔCT-method[4]. The housekeeping gene *ABL* and *GADPH* were used to normalize the data. The log2 fold change of all genes of interest compared to Nalm6 control cells was calculated. The procedure for Western blot was described in previous study.[5] Antibodies were listed in **Table S2**.

**Chromatin immunoprecipitation followed by sequencing (ChIP-Seq)**

The procedure was adapted from a previous study[6]. Approximately 1.10^8^ of Nalm6, REH or blasts from BCP-ALL patients were fixed in 1 % formaldehyde (#648336, Polysciences) at room temperature for 10 min and then quenched in 100 mM glycine for 1 min. Cells were washed twice in PBS and lysed 15 min in 50 mM Tris-HCl ph8.1/10 mM EDTA/0.5 % Empigen BB/1 % SDS. Lysates were sonicated to shear DNA to lengths between 200 and 600 base pairs and spun at 10,000 g for 10 min at 4°C. The sonicated cell supernatants were diluted 6-fold in ChIP Dilution Buffer (20 mM tris-HCl pH 8.1/150 mM NaCl/0.1 % triton) and incubated with 10 μg of anti-RUNX1 antibody or anti-ETV6 antibody or with 5 μg of anti-H3K4me3, anti-H3K4me1 or anti-H3K27ac overnight at 4°C. Antibodies used are: anti-RUNX1 antibody (#ab23980, Abcam, Cambridge, UK), anti-ETV6 antibody (#HPA000264, Sigma Aldrich, Missouri, USA), anti-H3K4me3 (#04-745, Merck Millipore, Massachusetts, USA), anti-H3K4me1 (#07-436, Merck Millipore) or anti-H3K27ac (#ab4729, Abcam). In the case of REH cells, RUNX1 antibodies will bind RUNX1 and ETV6-RUNX1, whereas ETV6 antibodies will bind ETV6-RUNX1 protein. Then, 100 µl of magnetic bead protein G (10004D, Invitrogen) and yeast tRNA (R5636, Sigma Aldrich) were added to the lysate for 4 h à 4°C under agitation. After four washings, immunoprecipitated DNA was eluted with elution buffer containing 1% SDS and 0.1 M NaHCO_3_. The protein–DNA crosslinks were reversed by heating at 65°C overnight, and chromatin was cleaned up using PCR purification kit (Qiagen). DNA quality was assessed with the Agilent Bioanalyzer with a High Sensitivity Chip. ChIP-Seq libraries were generated using TruSeq® ChIP Library Preparation Kit (Illumina) according to the manufacturer’s protocol. High-throughput sequencing was conducted on a Genome Analyzer II (Illumina, San Diego, CA) at the Human and Environmental Genomics' platform of Rennes (Biogenouest génomique, Rennes, France). ChIP-Seq reads were aligned to the reference human genome version GRCh37 (hg19) and peak calling were carried out as described previously[6]. All sequencing data are available at NCBI's Gene Expression Omnibus (GEO) (https://www.ncbi.nlm.nih.gov/geo/query) through GEO Series accession number GSE109377 for Nalm6 and BCP-ALL patients [7], GSE117684 for REH [8] and GSE176084 for REH ETV6-RUNX1 ChIP-Seq.

Tracks were visualized with Integrated Genome Browser 9.0.0[9].

**RUNX1 binding site identification**

Identification of RUNX1 potential binding sites was obtained using the Jaspar software tool (<http://jaspar.genereg.net/>)[10]. *RAG1* regions were scanned with the Jaspar’s human RUNX1 matrix (MA0002.1) and predicted sites with high sequence conservation (more than 80%) have been selected.

**Luciferase assay**

For luciferase test in Nalm6 cells, two chromatin regions of *RAG1* (corresponding to human hg19 chr11:36,589,391-36,589,603 and Chr11:36,588,180-36,588,525) were cloned with a minimal promoter (5’-AGACACTAGAGGGTATATAATGGAAGCTCGACTTCCAG-3’) into a *MSCV Luciferase PGK-hygro* vector (a gift from Scott Lowe, Addgene plasmid #18782) by the Gibson Assembly® Cloning Kit (NEB). To produce these cloned-MSCV luc retrovirus, HEK293 cells were co-transfected with 3.91 µg MSCV luc together with 1.23 µg pCMV-VSV-G (a gift from Bob Weinberg, Addgene plasmid #8454)[11] and with 3.91 µg pGag-pol[12] using Lipofectamine 3000 transfection reagent (Thermo Fisher Scientific). After 48h, supernatant was harvested, filtered, and added to Nalm6^control^, Nalm6^RUNX1^ and Nalm6^ETV6-RUNX1^ cells with 4 µg/mL of polybrene. Transduced cells were selected in medium containing 300 μg/mL hygromycin (Invivogen). Ten days after transduction, 1 million cells were seeded in duplicate in 100 µL of PBS into a MW96. Luciferase activities were determined upon addition of D-Luciferin (Cayman Chemicals) (1 mg/1 million cells) to the cells with a PhotonIMAGER™ Optima (Biospace Lab).

For luciferase test in HEK293 cells, the same chromatin regions of *RAG1* were cloned into *pGL4.10-luc* with a minimal promoter and transfected in HEK293 cells, in presence of *pFN-Halotag-RUNX1* or *pFN-Halotag-ETV6/RUNX1* and supplemented with empty *pCDNA* vector. Biological replicates of HEK293 cells were plated into 12-well plates and co-transfected with 0.25 μg of pGL4.10*-luc* plasmid DNA, together with 0.25 μg of the appropriate vector using lipofectamine 3000 (Thermo Fisher Scientific). *pCMV-renilla* luciferase vector was also transfected as an internal control for transfection efficiency. Forty-eight hours after transfection, cells were lysed and assayed for luciferase activity using the dual luciferase reporter system (Promega) according to the manufacturer's protocol and an LB 960 Centro luminometer (Berthold technologies).

**Chromatin immunoprecipitation - PCR (ChIP-PCR)**

We used the SimpleChIP Enzymatic Chromatin IP Kit (9002S, Cell signaling, Ozyme, France) and followed the recommendations of the manufacturer. Approximately 4$\times$10^6^ cells (Nalm6, Nalm6^RUNX1-Halotag^, Nalm6^ETV6-RUNX1-Halotag^, Nalm6^RUNX1-HA^, and Nalm6^RUNX1-HA+ETV6-RUNX1-Halotag^) were cross-linked in 1% formaldehyde (Thermo Scientific) for each immunoprecipitation. Cells were digested by micrococcal nuclease, then the nuclear pellet was suspended in chromatin immunoprecipitation (ChIP) buffer and sheared to a 150-900bp fragment size using the Covaris M200 sonicator with Duty Factor 5.0 and a total treatment time 16 min (8 min followed by 1min incubation in ice and 8 min again). The sheared chromatin was incubated with appropriate antibodies **(Table S2)** and H3 (Cell Signaling) as positive control, and normal rabbit IgG (Cell Signaling) as negative control. The forward (F) and reverse (R) primers of RAG1 promoter were described in **Table S1**. The binding capability was expressed as a percent of the total input chromatin.

**CRISPR-** **SAM mediated transcriptional activation**

The procedure was adapted from a previous study[13]. The following plasmids: lenti sgRNA (MS2)_zeo backbone (addgene #61427; http://n2t.net/addgene:61427; RRID:Addgene_61427) [13], EF1a-MS2-p65-HSF1-2A-Hygro-WPRE (addgene #89308; http://n2t.net/addgene:89308 ; RRID:Addgene_89308 ) [14], and lenti dCas9-VP64_Blast (addgene #61425; http://n2t.net/addgene:61425 ; RRID:Addgene_61425) [13] were gifts from Feng Zhang. Multiple sgRNAs targeting *RAG1* promoter and enhancer were cloned into lenti sgRNA (MS2)_zeo backbone following the SAM protocol, then all the cloned plasmids were verified by sequencing. The targeted sequences were for target-1200bp: sgRNA1: AGGTAAAGGCTGCATCTCCT; sgRNA2: CTCTCCCAGTGTTACTGCAG; sgRNA3 : GTGGCCCCAGGGCTGTTGTG, and for target-80bp: sgRNA1: TGTGGTTAGCCCTCCATGGT; sgRNA2: CAGCTGGAGCTGGGGTCTCC.

Nalm6 cell line stably expressing MS2-p65 and dCas9-VP64 was produced by lentiviral transduction. HEK293 cells were co-transfected with MS2-p65 or dCas9-VP64 together with *pSPAX2* and *pCMV-VSV-G* for packaging using Lipofectamin 3000 transfection reagent (Thermo Fisher Scientific). After 48h, supernatant was harvested, filtered, and added to Nalm6 cells with 4 µg/mL of polybrene. Transduced cells were selected in medium containing 400ug/ml Hygromycin (gibco) and 6 μg/mL Blasticidin (gibco). One week later, RNA of the stable cell line was extracted and RT-PCR was performed using primers for dCas9 and MS2-p65-HSF1 **(Table S1)**. Nalm6-dCas9-MS2 expressing cloned sgRNA was produced by transduct lenti-sgRNA into the Nalm6-dCas9-MS2 stable cell line using the same method above. 3 days after transduction, RNA and protein of these stable cell lines expressing different sgRNA were extracted for RT-PCR and Western Blot.

**Fluorescence-based quantitative RAG recombination activity Assay**

The GFPi-RFP construct and the cloning and analysis procedures were described in previous study.[15] GFPi carried consensus 12 and 23-RSS (recombination signal sequences) with heptamer CACAGTG and nonamer ACAAAAACC as descibed in [15]. GFPi-RFP lentiviral particles were generated by cotransfection of HEK293 cells with the pgag/pol, pVSV-g and MSCV-GFPi-IRES-RFP plasmid using Lipofectamin 3000 transfection reagent (Thermo Fisher Scientific). After 48h, supernatant was harvested, filtered, and added to Nalm6^control^, Nalm6^RUNX1^ and Nalm6^ETV6-RUNX1^ cells with 4 µg/mL of polybrene. Ten days after transduction, flow cytometry data was acquired with a BD Accuri^TM^C6 Flow cytometer. RFP was measured on FL3 and GFP was measured on FL1. All data was analysed using the FlowJo^TM^ V10 software. The test on HEK293 cells were performed by co-transfecting the HEK293 cells with MSCV-GFPi-IRES-RFP plasmid and Halotag-RUNX1 or Halotag-ETV6-RUNX1 or Halotag-control plasmid. One week after transfection, fluorescence was measured.

**Statistical analysis**

Statistical analyses were performed with GraphPad Prism 6.0 software. Student’s t test was used for statistical analysis unless otherwise indicated, with values of *p* <0.05 considered significant. Correlations were analyzed with Pearson test and linear regression was calculated. When applicable, the data are presented as the mean ± S.D., **p* <0.05, ** *p* <0.01, *** *p* <0.001, **** *p* <0.0001.

***Supplemental Table S1: List of primers used in this study.***

| Gene | Forward | Reverse |
| --- | --- | --- |
| RAG1 | AAAGCCCACCCTAAGTTTTCA | CACAGGACCATGGACTGGAT |
| RUNX1 | ACAAACCCACCGCAAGTC | CATCTAGTTTCTGCCGATGTCTT |
| ETV6-RUNX1 | AAGCCCATCAACCTCTCTCA | TCGTGGACGTCTCTAGAAGGA |
| dCas9 | AAGAGCAGACGGCTGGAAAAT | CTCTTGAAGTTGGGGGTCAGG |
| MS2 | TTACTCAGTTCGTGCTCGTGG | TGCCACCCCATTAGCGAAAT |
| RAG2 | AAGGCAGCTTGGAGTCTGAA | AGCGTCCTCCAAAGAGAACA |
| GAPDH | GAGTCAACGGATTTGGTCGT | TTGATTTTGGAGGGATCTCG |
| ABL | CCAAGAAGGGGCTGTCCT | ATGCTACTGGCCGCTGAA |
| RAG1 promoter-80bp | GGTCTCCTGGCCCATGATT | CTCTCTCCTTGCCCTCTTGT |
| RAG1 promoter-1200bp | AGCTCTCACCTGCCTTTCTC | ACTGGTCTGTGGCTTCTAGG |

***Supplemental Table S2: List of antibodies used.***

| Antigen | Reference | Uses |
| --- | --- | --- |
| RUNX1 | Ab23980 (abcam) | WB, ChIP-Seq, ChIP-qPCR |
| ETV6 | HPA00026(Sigma Aldrich) | ChIP-Seq |
| ETV6 | Ab54705 (abcam) | WB |
| RAG1 | Ab229673 (Abcam) | WB |
| Halotag | G9281 (Promega) | WB, ChIP-qPCR |
| HA | Ab9110 (Abcam) | WB, ChIP-qPCR |
| H3k4me3 | 04-745 (Merck Millipore) | ChIP-Seq |
| H3K27ac | ab4729 (Abcam) | ChIP-Seq |
| anti-H3K4me1 | 07-436(Merck Millipore) | ChIP-Seq |
| β-actin | Ab8226 (Abcam) | WB |
| HSC70 | #7298, clone B-6, (Santa Cruz) | WB |

**FIGURE LEGENDS FOR THE SUPPLEMENTAL INFORMATION**

**Figure S1. ETV6-RUNX1 and RUNX1 upregulate the expression of *RAG1* mRNA and protein.**

**A.** Statistical analysis of the expression between *RUNX1* and *RAG1* mRNA originating from childhood BCP-ALL cells negative for ETV6-RUNX1 using Pearson correlation. Data of mRNA levels (expressed in Fragments Per Kilobase Million – FPKM) have been extracted from the St. Jude Children's Research Hospital RNA-Seq Pediatric Cancer Data Portal[16]. It was previously demonstrated that *RAG1* transcripts are over-represented in *ETV6-RUNX1* BCP-ALL blasts compared to other childhood BCP-ALL blasts[17][18][19]. Here we show a significant correlation between *RUNX1* and *RAG1* mRNA in ETV6-RUNX1 BCP-ALL.

**B.** Densitometric analysis showing the quantitation of endogenous RAG1 protein in Nalm6^control^, Nalm6^ETV6-RUNX1^ and Nalm6^RUNX1^ cells. Results are presented after normalizing with HSC70 protein levels. Each value represents the mean ±S.D. of four independent experiments (illustrated in Figure 1C).

**C.** Relative mRNA expression of *RUNX1* and *RAG1* measured by RT-qPCR in Nalm6, Nalm6^RUNX1^ and Nalm6^truncated RUNX1^ cells. RUNX1 is here truncated in its RUNT domain. Results are presented in-terms of a fold change in log2 after normalizing with *GAPDH* mRNA. Each value represents the mean ±S.D. of six independent experiments.

**D.** Representative images of western blot (left panel) and densitometric analysis (right panel) showing the quantitation of endogenous RAG1 protein in Nalm6, Nalm6^RUNX1^ and Nalm6^truncated RUNX1^ cells. Results are presented after normalizing with β-actin protein levels. The western blot also shows the presence of RUNX1 and truncated-RUNX1 revealed with HA antibody. Each value represents the mean ±S.D. of four independent experiments.

**Figure S2: ETV6-RUNX1 and RUNX1 share common chromatin regions within the *RAG* locus.**

**A.** Venn-diagram presenting the intersection between RUNX1 binding regions in both replicates in REH cells, and ETV6-RUNX1 (ETV6 antibody) binding regions in both replicates in REH cells. The number of sites exclusively occupied by ETV6-RUNX1 are indicated in the green box. Of note, in REH cells, RUNX1 antibodies will bind RUNX1 and ETV6-RUNX1, whereas ETV6 antibodies will bind ETV6-RUNX1 protein.

**B.** Density heatmaps of H3K4me1, H3K4me3 and H3K27ac ChIP signal at RUNX1 peaks (left panel) or ETV6-RUNX1 peaks (right panel). H3K4me1 marks active enhancers, H3K4me3 active promoters and H3K27ac transcriptionally active chromatin.

**C.** ChIP-Seq profiles across the human *RAG* locus. Genomic tracks display ChIP-Seq profiles for RUNX1, ETV6-RUNX1 and the histones H3K27ac and H3K4me3 from REH cells (2 replicates for RUNX1 and ETV6-RUNX1). RUNX1 and H3K27Ac ChIP-Seq for Nalm6 cells and RUNX1 ChIP-Seq bone marrow mononuclear cells isolated from three pre-B acute lymphoblastic leukemia patients (BCP-ALL) are also displayed. Of note, in REH cells, RUNX1 antibodies will bind RUNX1 and ETV6-RUNX1, whereas ETV6 antibodies will specifically bind ETV6-RUNX1 protein. ChIP-Seq data were acquired by Illumina sequencing and visualized with Integrated Genome Browser 9.0.0[9]. ChIP-Seq reads were aligned to the reference human genome version GRCh37 (hg19). Boxes show common *RAG* locus regulatory regions previously described in T- or B-cell lineages. Among them, in mouse T-cells, transcriptional regulation of *Rag1* and *Rag2* genes involves *cis-*regulatory elements including the *Rag2* promoter, *Rag1* promoter, a -2.6kb *Rag2* enhancer (also named the proximal enhancer, Ep) and a -8kb *Rag2* enhancer (also named the distal enhancer, Ed), a -85kb AntiSilencer Element (ASE) and a *Rag1-Rag2* intergenic silencer[20][21][22][23][24][25]. In B-cells, *Rag1* and *Rag2* genes are controlled by the strong -22kb *Erag* enhancer[21][26][22], the *Irag2* enhancer located close to *Erag* [27], *Rag2* distal and proximal enhancers (Ed and Ep), *Rag1* and *Rag2* promoter [28][29][20][30] [22][31] and *Irag1* located about 15 kb upstream of the Rag1 promoter[27].

**Figure S3. ETV6-RUNX1 and RUNX1 compete for the binding on the enhancer and the promoter of *RAG1*.**

**A.** ChIP-qPCR on the -1200 bp enhancer and the -80bp promoter with IgG and RUNX1 antibodies in Nalm6 cells. Results are expressed as percentage of input.

**B-E.** ChIP-qPCR on the -1200 bp enhancer (B-C) and the -80bp promoter (D-E) with IgG, HA and Halotag antibodies in Nalm6^RUNX1-HA^, Nalm6^RUNX1-HA+ETV6/RUNX1^, Nalm6^ETV6-RUNX1-Halotag^, and Nalm6^ETV6-RUNX1-Halotag+RUNX1^ cells. Results are expressed as percentage of input (n=3). On the -1200 bp enhancer, addition of ETV6-RUNX1 decreases by half the binding of RUNX1 (B). On the -80bp promoter, addition of RUNX1 clearly diminishes ETV6-RUNX1 binding (E).

**Figure S4. ETV6-RUNX1 and RUNX1 physiologically activate the transcription of *RAG1* by binding and increase RAG-mediated recombination.**

**A.** Relative mRNA expression of *RAG1* measured by RT-qPCR in HEK^control^, HEK transiently transfected with -1200gRNA plasmids (3 different sequences #1 to #3) and -80gRNA plasmids (2 different sequences #1 and #2) cells for the CRISPR dCas9-VP64 experiments. Results are presented in-terms of a fold change in log2 after normalizing with *GAPDH* mRNA. Each value represents the mean ±S.D. of three independent experiments.

**B.** Luciferase assays with plasmids containing either the -1200bp enhancer (left panel) or the -80bp promoter (right panel) of *RAG1* upstream a minimal promoter and a luciferase ORF, in presence of RUNX1 and ETV6-RUNX1 expressing vectors in HEK cells. Plasmid containing deleted RUNX1 consensus motif have also been tested. Luciferase levels are represented using a scatter dot plot indicating the means and S.D. of at least 4 independent experiments. ns: non-significant

**C.** Illustration of flow cytometry acquisition of RAG-mediated recombination using the reporter assay from [32] in Nalm6^control^, Nalm6^RUNX1^ and Nalm6^ETV6-RUNX1^ cells, 7 days after transduction of GFPi vectors. RAG-mediated recombination activity is mirrored by the percentage of GFP-positive cells in RFP-positive (gated) cells (n=4) (*i.e.* the top right quadrant of gated RFP+ cells (second line)). Quantitative results are given in **Figure 2E**.

**REFERENCES FOR THE SUPPLEMENTAL INFORMATION**

[1] L. Debaize, H. Jakobczyk, A.-G. Rio, V. Gandemer, and M.-B. Troadec, “Optimization of proximity ligation assay (PLA) for detection of protein interactions and fusion proteins in non-adherent cells: application to pre-B lymphocytes,” *Mol Cytogenet*, vol. 10, p. 27, 2017, doi: 10.1186/s13039-017-0328-2.

[2] D. Bluteau *et al.*, “Down-regulation of the RUNX1-target gene NR4A3 contributes to hematopoiesis deregulation in familial platelet disorder/acute myelogenous leukemia,” *Blood*, vol. 118, no. 24, pp. 6310–6320, Dec. 2011, doi: 10.1182/blood-2010-12-325555.

[3] M.-P. Arnaud *et al.*, “CD9, a key actor in the dissemination of lymphoblastic leukemia, modulating CXCR4-mediated migration via RAC1 signaling,” *Blood*, vol. 126, no. 15, pp. 1802–1812, Oct. 2015, doi: 10.1182/blood-2015-02-628560.

[4] J. H. Schefe, K. E. Lehmann, I. R. Buschmann, T. Unger, and H. Funke-Kaiser, “Quantitative real-time RT-PCR data analysis: current concepts and the novel ‘gene expression’s CT difference’ formula,” *J. Mol. Med.*, vol. 84, no. 11, pp. 901–910, Nov. 2006, doi: 10.1007/s00109-006-0097-6.

[5] H. Jakobczyk *et al.*, “Reduction of RUNX1 transcription factor activity by a CBFA2T3-mimicking peptide: application to B cell precursor acute lymphoblastic leukemia,” *J Hematol Oncol*, vol. 14, no. 1, p. 47, Mar. 2021, doi: 10.1186/s13045-021-01051-z.

[6] A. A. Sérandour *et al.*, “Dynamic hydroxymethylation of deoxyribonucleic acid marks differentiation-associated enhancers,” *Nucleic Acids Res.*, vol. 40, no. 17, pp. 8255–8265, Sep. 2012.

[7] L. Debaize *et al.*, “Interplay between transcription regulators RUNX1 and FUBP1 activates an enhancer of the oncogene c-KIT and amplifies cell proliferation,” *Nucleic Acids Res*, vol. 46, no. 21, pp. 11214–11228, Nov. 2018, doi: 10.1093/nar/gky756.

[8] H. Jakobczyk *et al.*, “Reduction of RUNX1 transcription factor activity by a CBFA2T3-mimicking peptide: application to B cell precursor acute lymphoblastic leukemia,” *J Hematol Oncol*, vol. 14, no. 1, p. 47, Mar. 2021, doi: 10.1186/s13045-021-01051-z.

[9] J. W. Nicol, G. A. Helt, S. G. Blanchard, A. Raja, and A. E. Loraine, “The Integrated Genome Browser: free software for distribution and exploration of genome-scale datasets,” *Bioinformatics*, vol. 25, no. 20, pp. 2730–2731, Oct. 2009, doi: 10.1093/bioinformatics/btp472.

[10] A. Sandelin, W. Alkema, P. Engström, W. W. Wasserman, and B. Lenhard, “JASPAR: an open-access database for eukaryotic transcription factor binding profiles,” *Nucleic Acids Res.*, vol. 32, no. Database issue, pp. D91-94, Jan. 2004, doi: 10.1093/nar/gkh012.

[11] S. A. Stewart *et al.*, “Lentivirus-delivered stable gene silencing by RNAi in primary cells,” *RNA*, vol. 9, no. 4, pp. 493–501, Apr. 2003.

[12] D. Nègre *et al.*, “Characterization of novel safe lentiviral vectors derived from simian immunodeficiency virus (SIVmac251) that efficiently transduce mature human dendritic cells,” *Gene Ther.*, vol. 7, no. 19, pp. 1613–1623, Oct. 2000, doi: 10.1038/sj.gt.3301292.

[13] S. Konermann *et al.*, “Genome-scale transcriptional activation by an engineered CRISPR-Cas9 complex,” *Nature*, vol. 517, no. 7536, pp. 583–588, Jan. 2015, doi: 10.1038/nature14136.

[14] J. Joung *et al.*, “Genome-scale CRISPR-Cas9 knockout and transcriptional activation screening,” *Nat Protoc*, vol. 12, no. 4, pp. 828–863, Apr. 2017, doi: 10.1038/nprot.2017.016.

[15] I. Trancoso *et al.*, “A Novel Quantitative Fluorescent Reporter Assay for RAG Targets and RAG Activity,” *Front Immunol*, vol. 4, May 2013, doi: 10.3389/fimmu.2013.00110.

[16] X. Zhou *et al.*, “Exploring genomic alteration in pediatric cancer using ProteinPaint,” *Nat. Genet.*, vol. 48, no. 1, pp. 4–6, Jan. 2016, doi: 10.1038/ng.3466.

[17] E.-J. Yeoh *et al.*, “Classification, subtype discovery, and prediction of outcome in pediatric acute lymphoblastic leukemia by gene expression profiling,” *Cancer Cell*, vol. 1, no. 2, pp. 133–143, Mar. 2002.

[18] M. E. Ross *et al.*, “Classification of pediatric acute lymphoblastic leukemia by gene expression profiling,” *Blood*, vol. 102, no. 8, pp. 2951–2959, Oct. 2003, doi: 10.1182/blood-2003-01-0338.

[19] M. Heinaniemi *et al.*, “Transcription-coupled genetic instability marks acute lymphoblastic leukemia structural variation hotspots,” *eLife*, vol. 5, p. e13087, Jul. 2016, doi: 10.7554/eLife.13087.

[20] G. A. Miranda *et al.*, “Combinatorial regulation of the murine RAG-2 promoter by Sp1 and distinct lymphocyte-specific transcription factors,” *Mol. Immunol.*, vol. 38, no. 15, pp. 1151–1159, Jun. 2002.

[21] L.-Y. Hsu *et al.*, “A conserved transcriptional enhancer regulates RAG gene expression in developing B cells,” *Immunity*, vol. 19, no. 1, pp. 105–117, Jul. 2003.

[22] T. C. Kuo and M. S. Schlissel, “Mechanisms controlling expression of the RAG locus during lymphocyte development,” *Curr. Opin. Immunol.*, vol. 21, no. 2, pp. 173–178, Apr. 2009, doi: 10.1016/j.coi.2009.03.008.

[23] N. Yannoutsos *et al.*, “A cis element in the recombination activating gene locus regulates gene expression by counteracting a distant silencer,” *Nat. Immunol.*, vol. 5, no. 4, pp. 443–450, Apr. 2004, doi: 10.1038/ni1053.

[24] B. Hao *et al.*, “An anti-silencer- and SATB1-dependent chromatin hub regulates Rag1 and Rag2 gene expression during thymocyte development,” *J. Exp. Med.*, vol. 212, no. 5, pp. 809–824, May 2015, doi: 10.1084/jem.20142207.

[25] A. K. Naik, A. T. Byrd, A. C. K. Lucander, and M. S. Krangel, “Hierarchical assembly and disassembly of a transcriptionally active RAG locus in CD4+CD8+ thymocytes,” *J Exp Med*, vol. 216, no. 1, pp. 231–243, Jan. 2019, doi: 10.1084/jem.20181402.

[26] H. Hu *et al.*, “Foxp1 is an essential transcriptional regulator of B cell development,” *Nature Immunology*, vol. 7, no. 8, Art. no. 8, Aug. 2006, doi: 10.1038/ni1358.

[27] K. Ochiai *et al.*, “A self-reinforcing regulatory network triggered by limiting interleukin-7 activates pre-BCR signaling and differentiation,” *Nat Immunol*, vol. 13, no. 3, pp. 300–307, Jan. 2012, doi: 10.1038/ni.2210.

[28] K. Fuller and U. Storb, “Identification and characterization of the murine Rag1 promoter,” *Mol. Immunol.*, vol. 34, no. 12–13, pp. 939–954, Sep. 1997.

[29] H. Kishi *et al.*, “Lineage-specific regulation of the murine RAG-2 promoter: GATA-3 in T cells and Pax-5 in B cells,” *Blood*, vol. 95, no. 12, pp. 3845–3852, Jun. 2000.

[30] X.-C. Wei, J. Dohkan, H. Kishi, C.-X. Wu, S. Kondo, and A. Muraguchi, “Characterization of the proximal enhancer element and transcriptional regulatory factors for murine recombination activating gene-2,” *European Journal of Immunology*, vol. 35, no. 2, pp. 612–621, Feb. 2005, doi: 10.1002/eji.200425185.

[31] B.-S. Lee *et al.*, “Corrected and Republished from: BCL11A is a critical component of a transcriptional network that activates RAG expression and VDJ recombination,” *Mol. Cell. Biol.*, 16 2017, doi: 10.1128/MCB.00362-17.

[32] I. Trancoso *et al.*, “A Novel Quantitative Fluorescent Reporter Assay for RAG Targets and RAG Activity,” *Front Immunol*, vol. 4, May 2013, doi: 10.3389/fimmu.2013.00110.
